# The impact of sphygmomanometer placement and cuff placement on blood pressure measurements

**DOI:** 10.3389/fcvm.2024.1388313

**Published:** 2024-06-18

**Authors:** Xiao-Yong Zhu, Pu-Hua Zhang, Wen-Yin Huang, Wan Huang, Xin-Hu Tang, Hua Yu, Su-Nan Wang

**Affiliations:** Department of Cardiology, Jiujiang University Affiliated Hospital, Jiujiang, Jiangxi Province, China

**Keywords:** blood pressure, cardiovascular disease, hypertension, electronic sphygmomanometer, mercury sphygmomanometer

## Abstract

**Background:**

Hypertension is the most significant global risk factor for mortality and morbidity, making standardized blood pressure measurement crucial.

**Objectives:**

To investigate whether the location of blood pressure monitors and the positioning of cuffs yield differing results in blood pressure measurements.

**Methods:**

Patients admitted to the Affiliated Hospital of Jiujiang College between 1 January 2022 and 30 June 2023 were enrolled in this study and randomly allocated into four groups. These groups were defined based on the positioning of monitoring equipment as follows: varied placements of cuffs on automatic blood pressure monitors, different heights for mercury column blood pressure monitors, varied heights for automatic blood pressure monitors, and different orientations for the cuff airbag tubes on electrocardiogram monitors. Blood pressure was measured and recorded for each group, followed by an analysis of the variations in readings across the different setups.

**Results:**

In the first cohort of 763 individuals, mean systolic blood pressure measured at the standard upper arm site was 128.8 ± 10.5 mmHg, compared to 125.3 ± 10.4 mmHg at the elbow fossa. The corresponding diastolic pressures were 79.2 ± 10.7 and 75.0 ± 10.6 mmHg, respectively. The difference in systolic pressure between these positions was significant at 3.48 ± 3.22 mmHg (*t*₁ = 29.91, *p*₁ < 0.001) and for diastolic pressure at 4.23 ± 1.31 mmHg (*t*₂ = 88.98, *p*₂ < 0.001). For the subsequent groups, involving 253, 312, and 225 individuals, respectively, blood pressure measurements were analyzed and compared across different methods within each group. All *p*-values exceeded 0.05, indicating no statistically significant differences.

**Conclusions:**

Blood pressure values measured at the elbow fossa position using an upper arm-type automatic sphygmomanometer were found to be lower than those measured at the upper arm position, with a difference of 3.48 mmHg for systolic and 4.23 mmHg for diastolic pressures. It is therefore essential to position the cuff correctly, specifically 2–3 cm above the elbow fossa, when utilizing an upper arm-type automatic sphygmomanometer for blood pressure monitoring. Conversely, the placement of the mercury column sphygmomanometer and the automated sphygmomanometer at varying heights had no significant effect on blood pressure readings. Similarly, the orientation of the electrocardiogram's cuffed balloon tube, whether facing upward or downward, did not influence blood pressure measurement outcomes.

## Introduction

Cardiovascular diseases are the leading cause of death globally (1). Hypertension is a major risk factor for mortality and disability worldwide, affecting over one billion individuals and contributing to an estimated 9.4 million deaths each year (2). Regular monitoring of blood pressure is crucial, and blood pressure can be measured by auscultation or automated oscillometric methods. Timely adjustment of antihypertensive treatment based on blood pressure levels can significantly reduce the risk of cardiovascular events (3). However, the accuracy of blood pressure measurements can be compromised by various factors, which may not reflect the patient's actual blood pressure accurately, such as the duration of rest before measurement (4), and the elasticity of the cuff during measurement (5). Inaccurate blood pressure readings could potentially lead to missed opportunities for reducing cardiovascular risk or to the unnecessary intensification of medication. As such, it is important to adhere to standardized measurement techniques to enhance the accuracy of blood pressure readings.

## Methods

The data supporting the findings of this study are available from the corresponding author upon reasonable request.

### Randomization and design

**Figure 1:**
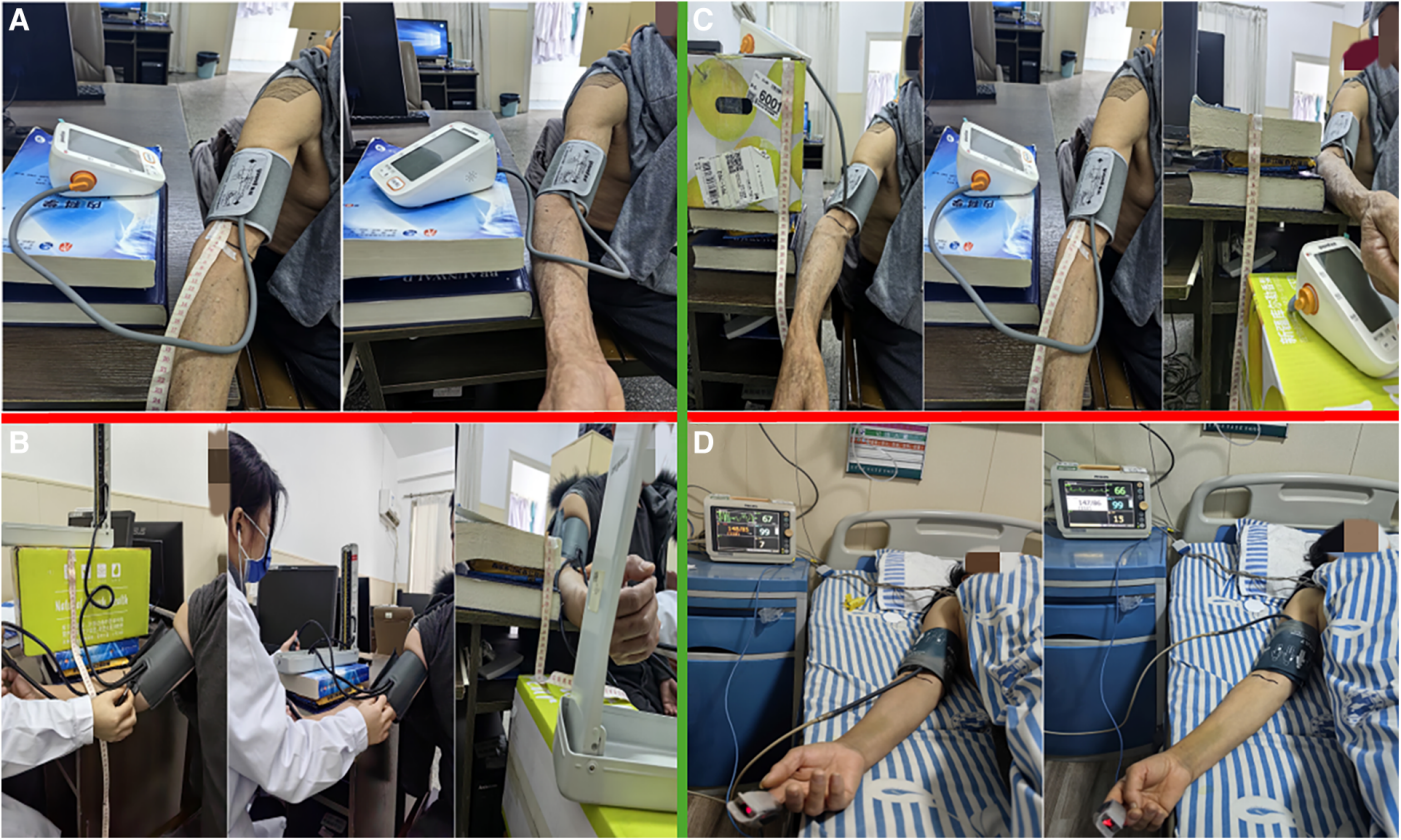
Four different sets of methods to perform blood pressure measurements. (**A**) Used the same upper arm sphygmomanometer to measure blood pressure and heart rate at the standard upper arm level and the elbow fossa position; (**B**) Positioned the zero scale level of mercury sphygmomanometers at 20 cm above, at the same level (0 cm), and 20 cm below the heart level to measure and record blood pressure readings; (**C**) Positioned the zero scale level of electronic sphygmomanometers at 20 cm above, at the same level (0 cm), and 20 cm below the heart level to measure and record blood pressure readings; (**D**) Conducted blood pressure measurements with the cuff airbag tube of the cardiac monitor facing both upward and downward.

To investigate the effects of sphygmomanometer placement and cuff position on blood pressure measurement, we conducted a series of randomized controlled experiments divided into four groups:

Group A used the same upper arm sphygmomanometer to measure blood pressure and heart rate at the standard upper arm level and the elbow fossa position, recording the results separately. This was to explore the effects of cuff placement on the measurements obtained by electronic sphygmomanometers.
Group B positioned the zero scale level of mercury sphygmomanometers at 20 cm above, at the same level (0 cm), and 20 cm below the heart level to measure and record blood pressure readings. This group aimed to determine the impact of the mercury sphygmomanometer's placement on blood pressure readings.
Group C positioned the zero scale level of electronic sphygmomanometers at 20 cm above, at the same level (0 cm), and 20 cm below the heart level to measure and record blood pressure readings. This group aimed to determine the impact of the electronic sphygmomanometer's placement on blood pressure readings.
Group D conducted blood pressure measurements with the cuff airbag tube of the cardiac monitor facing both upward and downward, recording the results to assess whether the orientation of the airbag tube affected the blood pressure readings (refer to Figure 1).

Self-paired tests were administered, with the results categorized, registered, and subjected to statistical analysis. The Institutional Review Board of Jiujiang College Hospital approved the study protocol. All study participants provided written informed consent.

### Study population

The inclusion criteria consisted of the following: patients hospitalized at the Affiliated Hospital of Jiujiang College, age 18 years or older, an upper arm circumference between 22 and 34 cm, stable vital signs, and ability to cooperate with the blood pressure measurement process.

Participants were primarily excluded based on conditions that could affect blood pressure measurements, including the following: rash, edema, paralysis, open ulcers or wounds, or arteriovenous shunts in both arms; atrial fibrillation, cardiac arrhythmia, or hemodynamic instability; recent phlebotomy; cognitive disorders; pregnancy; and Hb levels below 60 g/L.

### Data collection

Collected data included age, gender, height, weight, body mass index (BMI), measured systolic and diastolic blood pressures, and heart rate levels of the individuals.

### Blood pressure measurements

Three types of devices were utilized: (1) a standard mercury sphygmomanometer (reference device: Yuwell sphygmomanometer), also referred to as an “auscultation device” (Jiangsu Yuyue Medical Equipment Co., Ltd., Jiangsu, China); (2) an Omron oscillometric device (HEM-7121; Omron Healthcare, Kyoto, Japan); and (3) a Philips cardiac monitor (SureSigns VM6; Royal Philips, Amsterdam, Netherlands). The sphygmomanometers were verified to be in good condition and working order. Before measurement, the devices were sent to the hospital's medical engineering department for testing and calibration, with each component's function being checked and approved.

The measurement technique for each device adhered to the standard recommendations for blood pressure measurements and each manufacturer's instructions, as well as the 2017 American College of Cardiology (ACC)/American Heart Association (AHA) Hypertension Guidelines (6). Participants were required to refrain from strenuous activities and avoid consuming coffee or alcohol for 30 min before blood pressure measurement. After emptying their bladders, they sat quietly for 5–10 min. Each participant remained comfortably seated with their back supported by the chair, did not talk during the measurement, and fully exposed their right upper arm. The center of the cuff was aligned with the level of the patient's right atrium (at the midpoint of the sternum) (6). Blood pressure was measured at intervals of 1–2 min, with three consecutive measurements taken; the average of these three measurements was recorded. If the difference between any two systolic or diastolic measurements exceeded 5 mmHg, a fourth measurement was taken, and the average of the four measurements was recorded.

### Statistical analysis

Data were processed and analyzed using Excel 2019 and SPSS version 27.0 software. The normality of the distribution was assessed using the Shapiro–Wilk test. Measurement data conforming to a normal distribution were expressed as the mean ± standard deviation ($\bar{x}$ *± s*). Comparisons between two groups were made using the paired-sample *t*-test. Count data were expressed as the number of instances (%) and agreement was analyzed using the Bland–Altman method. A difference of *p* < 0.05 was considered statistically significant.

## Results

The first experimental group consisted of 763 individuals with a mean age of 57.5 ± 10.1 years (446 men, 58.5%); their mean height was 167.6 ± 8.3 cm, weight was 62.1 ± 10.5 kg, and BMI was 22.0 ± 2.8 kg/m^2^.

Table 1 presents the blood pressure and heart rate values measured by the participants in the upper arm and elbow fossa positions. The mean systolic and diastolic blood pressure readings in the upper arm position were higher than those in the elbow fossa position for all individuals (both *p* < 0.001). The mean difference in systolic blood pressure between the upper arm and the elbow fossa positions was 3.48 ± 3.2 mmHg, and the mean difference in diastolic blood pressure was 4.23 ± 1.31 mmHg. There was no significant difference in heart rate between the upper arm and the elbow fossa positions (*p* = 0.147).

**Table 1:** Blood pressure measurements x at upper arm and elbow fossa positions $\bar{x}$_ _± s (a paired-sample *t*-test was used).

Population	Number of cases	Measurement site	SBP (mmHg)	DBP (mmHg)	Heart rate (beats/min)
All	763	Upper arm position	128.8 ± 10.5	79.2 ± 10.7	72.8 ± 9.1
		Elbow fossa position	125.3 ± 10.4	75.0 ± 10.6	73.0 ± 8.2
		Difference	3.48 ± 3.22	4.23 ± 1.31	−0.20 ± 3.77
		95% CI	3.26–3.71	4.13–4.32	−0.47 to 0.07
		*t*	29.91	88.98	−1.46
		*p*	<0.001	<0.001	0.147

The difference is the ratio of upper arm position to elbow fossa position, *t*-value is the ratio of upper arm position to elbow fossa position, and *p*-value is the ratio of upper arm position to elbow socket position.

**Figure 2:**
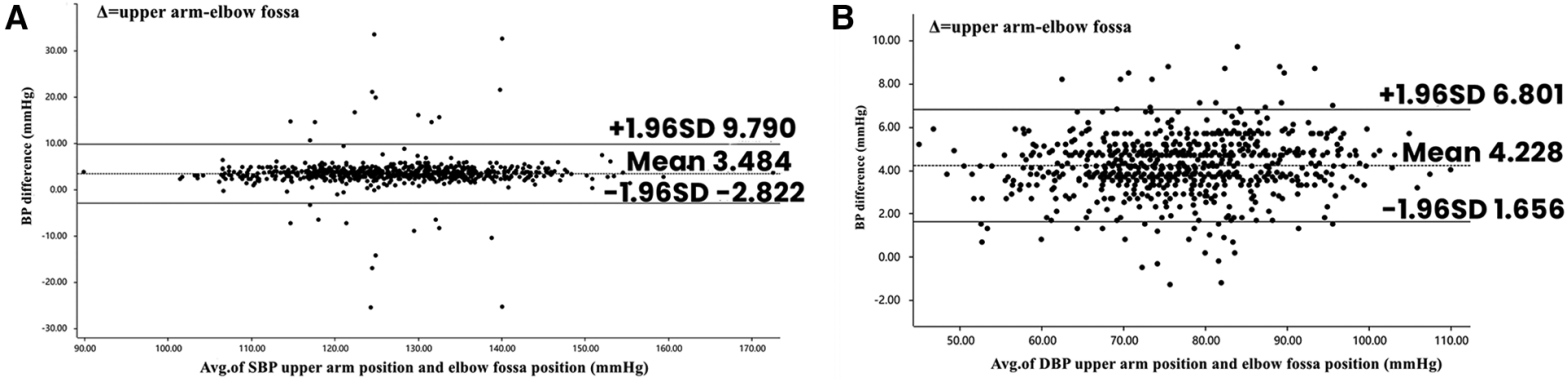
Bland–Altman plots depicting the degree of BP differences across the range of BP between elbow fossa position and upper arm position. (**A**) The difference in systolic BP between upper arm position and elbow fossa position in electronic sphygmomanometer. (**B**) The difference in diastolic BP between upper arm position and elbow fossa position in electronic sphygmomanometer. BP, blood pressure.

Figure 2 displays the Bland–Altman analysis of systolic and diastolic blood pressure measurements taken with an electronic blood pressure monitor in the standard upper arm and elbow fossa positions (*n* = 763).

The second experimental group included 253 participants with a mean age of 58.4 ± 9.3 years (156 men, 61.7%); their mean height was 169.1 ± 5.9 cm, weight was 64.3 ± 8.5 kg, and BMI was 23.0 ± 2.7 kg/m^2^.

**Figure 3:**
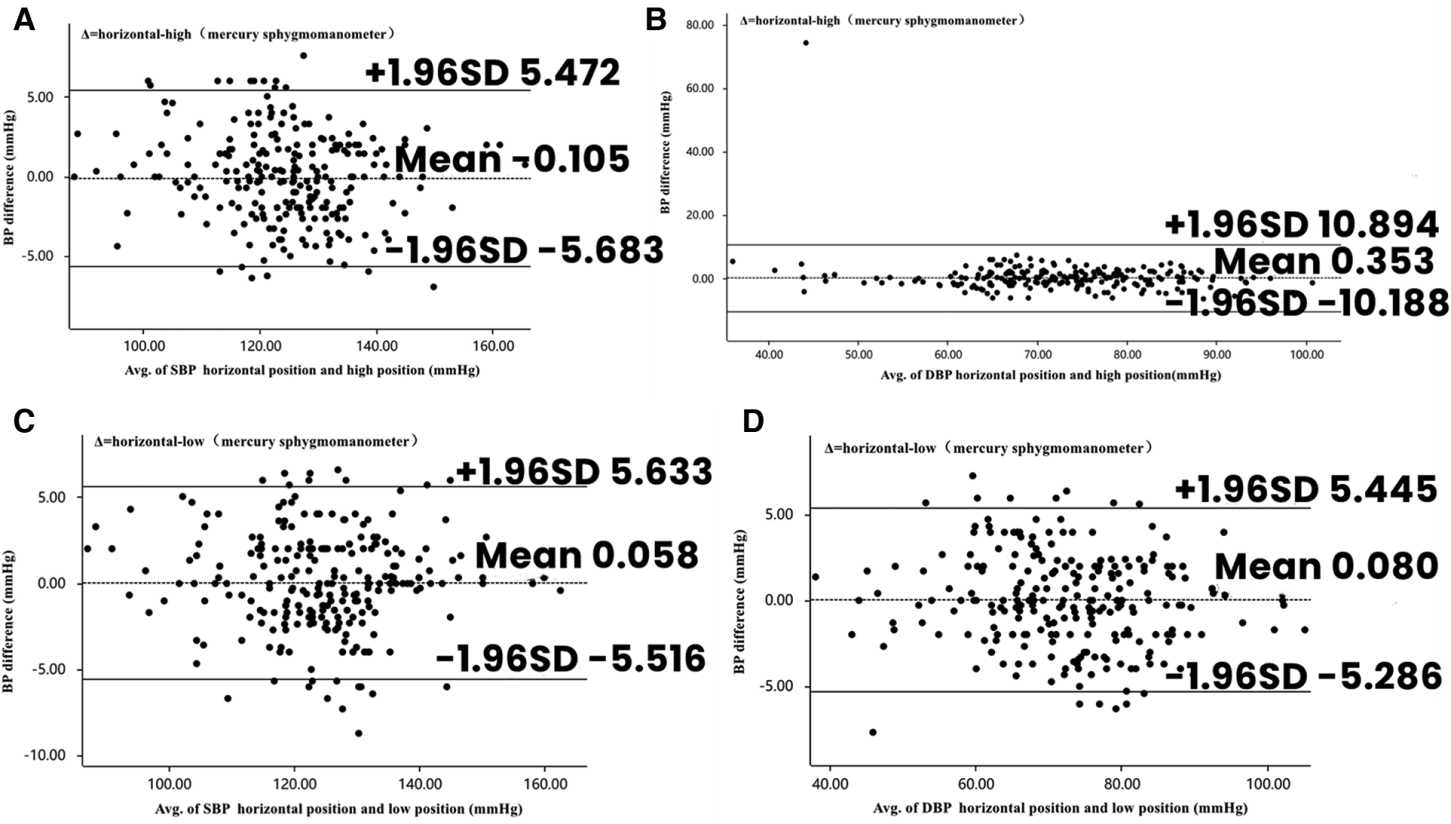
Bland–Altman plots depicting the degree of BP differences across the range of BP in placement of mercury sphygmomanometers in different positions. (**A**) The difference in SBP between horizontal and high positions in mercury sphygmomanometers. (**B**) The difference in DBP between horizontal and high positions in mercury sphygmomanometers. (**C**) The difference in SBP between horizontal and low positions in mercury sphygmomanometers. (**D**) The difference in DBP between horizontal and low positions in mercury sphygmomanometers. BP, blood pressure; DBP, diastolic blood pressure; SBP, systolic blood pressure.

Table 2 shows the blood pressure readings of the participants measured by a mercury sphygmomanometer at three different sites. There were no significant differences in systolic (*p* = 0.557) and diastolic (*p* = 0.297) blood pressure between the horizontal and high positions for all participants. Similarly, there were no significant differences in systolic (*p* = 0.744) and diastolic (*p* = 0.643) blood pressure between the horizontal and low positions for all participants. Figure 3 depicts the Bland–Altman analysis of systolic and diastolic blood pressure measurements taken with the mercury sphygmomanometer in different positions (*n* = 253).

**Table 2:** Blood pressure measurements of mercury sphygmomanometer in high, horizontal, and low positions $\bar{x}$_ _± s, using a paired samples *t*-test.

Population	Number of cases	Measurement site	SBP (mmHg)	DBP (mmHg)
All	253	High positions	124.89 ± 12.78	72.30 ± 12.01
		Horizontal positions	124.79 ± 12.31	72.66 ± 11.16
		Low positions	124.73 ± 12.42	72.58 ± 11.53
		Difference 1	−0.11 ± 2.85	0.35 ± 5.38
		Difference 2	0.06 ± 2.84	0.08 ± 2.73
		95% CI 1	−0.46 to 0.25	−0.31 to 1.02
		95% CI 2	−0.29 to 0.41	−0.26 to 0.42
		*t*1	−0.588	1.045
		*t*2	0.327	0.464
		^*P*	NS	NS
		#*P*	NS	NS

Difference 1, difference in blood pressure between horizontal and high position; Difference 2, difference in blood pressure between horizontal and low position; 95% CI 1, confidence interval for the difference between horizontal and high position; 95% CI 2, confidence interval for the difference between horizontal and low position; *t*1, horizontal compared to high position; *t*2, horizontal compared to low position; ^P, horizontal compared to high position; #P, horizontal compared to low position.

The third experimental group comprised 312 participants with a mean age of 65.68 ± 11.4 years (167 men, 53.5%); their mean height was 166.0 ± 6.9 cm, weight was 61.6 ± 9.2 kg, and BMI was 22.3 ± 2.4 kg/m^2^.

Table 3 indicates the blood pressure readings of the participants measured by an electronic sphygmomanometer at three different sites. There were no significant differences in systolic (*p* = 0.362) and diastolic (*p* = 0.174) blood pressures between the horizontal and high positions for all participants. Furthermore, there were no significant differences in systolic (*p* = 0.222) and diastolic (*p* = 0.271) blood pressures between the horizontal and low positions for all participants. No significant differences were observed in heart rate (*p* = 0.445) between the horizontal and high positions for all participants; there was no significant difference between horizontal (*p* = 0.313).

**Table 3:** Blood pressure measurements of electronic sphygmomanometers in high, horizontal, and low positions $\bar{x}$_ _± s, using a paired samples *t*-test.

Population	Number of cases	Measurement site	SBP (mmHg)	DBP (mmHg)	Heart rate (beats/min)
All	312	High positions	122.47 ± 14.94	73.80 ± 10.27	75.05 ± 7.61
		Horizontal positions	122.61 ± 14.69	73.60 ± 9.92	74.89 ± 6.70
		Low positions	122.78 ± 14.70	73.45 ± 10.21	75.03 ± 7.08
		Difference 1	0.13 ± 2.56	−0.20 ± 2.56	−0.16 ± 3.78
		Difference 2	−0.18 ± 2.57	0.16 ± 2.54	−0.14 ± 2.52
		95% CI 1	−0.15 to 0.42	−0.48 to 0.09	−0.58 to 0.26
		95% CI 2	−0.46 to 0.11	−0.12 to 0.44	−0.43 to 0.14
		*t*1	0.913	−1.363	0.765
		*t*2	−1.223	1.102	−1.010
		^P	NS	NS	NS
		#P	NS	NS	NS

Difference 1, difference in blood pressure between horizontal and high position; Difference 2, difference in blood pressure between horizontal and low position; 95% CI 1, confidence interval for the difference between horizontal and high position; 95% CI 2, confidence interval for the difference between horizontal and low position; *t*1, horizontal compared to high position; *t*2, horizontal compared to low position; ^P, horizontal compared to high position; #P, horizontal compared to low position.

**Figure 4:**
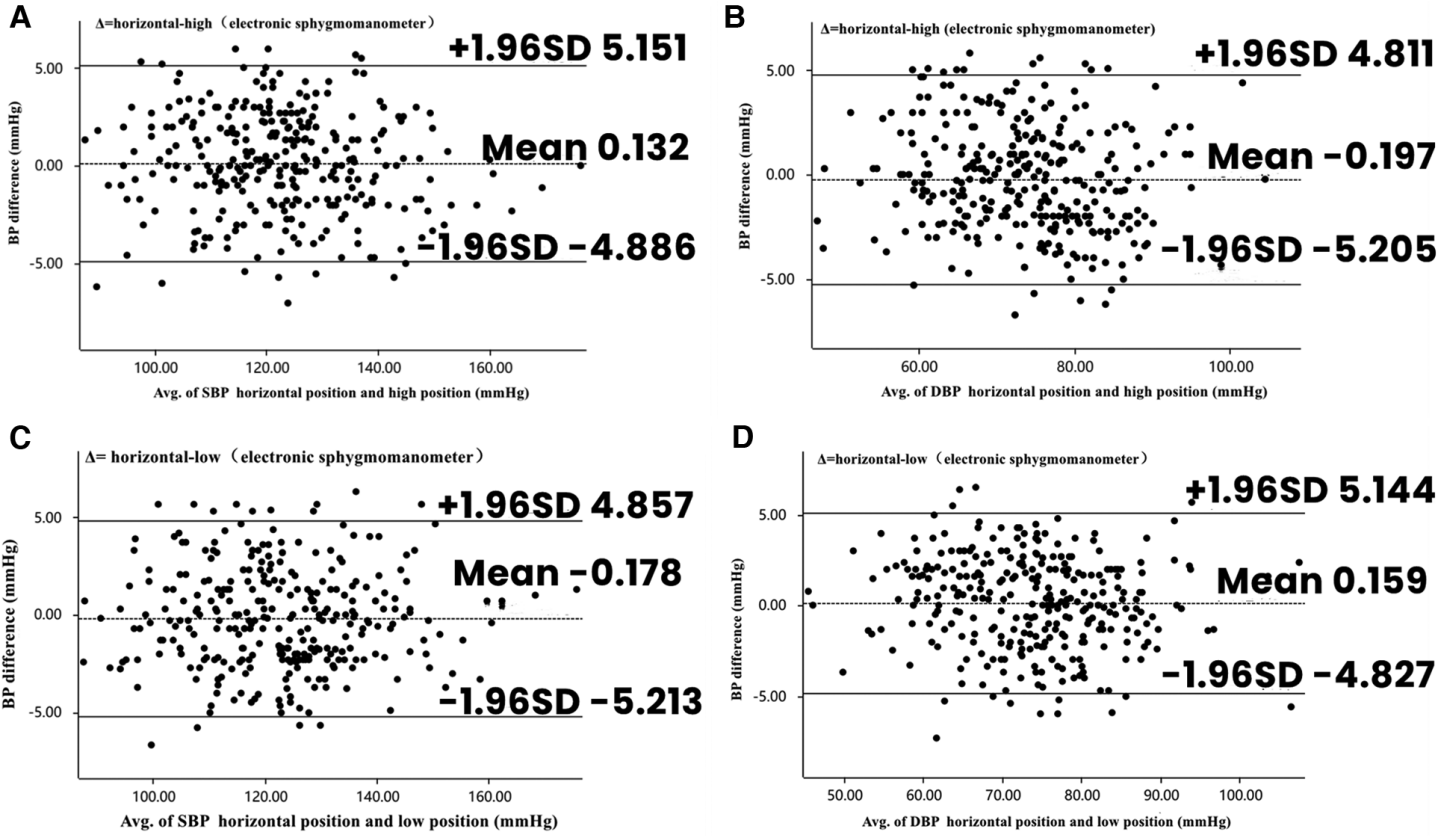
Bland–Altman plots depicting the degree of BP differences across the range of BP in placement of electronic sphygmomanometers in different positions. (**A**) The difference in SBP between horizontal and high positions in electronic sphygmomanometers. (**B**) The difference in DBP between horizontal and high positions in electronic sphygmomanometers. (**C**) The difference in SBP between horizontal and low positions in electronic sphygmomanometers. (**D**) The difference in DBP between horizontal and low positions in electronic sphygmomanometers. BP, blood pressure; DBP, diastolic blood pressure; SBP, systolic blood pressure.

Figure 4 illustrates the Bland–Altman analysis of systolic and diastolic blood pressure measurements taken with the electronic sphygmomanometer in different positions (*n* = 312).

The fourth experimental group included 225 participants with a mean age of 62.2 ± 8.5 years (132 men, 58.7%); their mean height was 166.8 ± 6.7 cm, weight was 63.9 ± 10.1 kg, and BMI was 22.9 ± 2.6 kg/m^2^.

**Figure 5:**
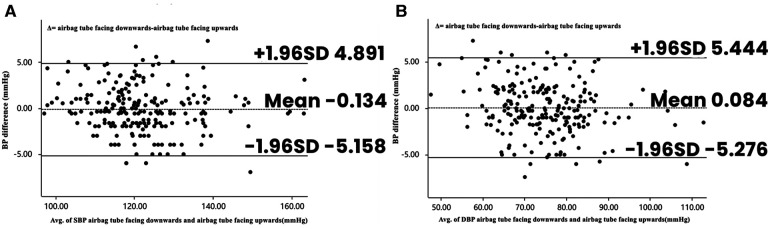
Bland–Altman plots depicting the degree of BP differences across the range of BP with the cuff airbag tube facing downward and upward. (**A**) The difference in SBP between ECG monitor cuff airbag tube facing downward and facing upward. (**B**) The difference in DBP between ECG monitor cuff airbag tube facing downward and facing upward. BP, blood pressure; DBP, diastolic blood pressure; SBP, systolic blood pressure.

Table 4 demonstrates the blood pressure and heart rate values measured by the sphygmomanometers when the participants had the cardiac monitor cuff with the balloon tubes facing down and up. There were no significant differences in systolic (*p* = 0.435) or diastolic (*p* = 0.645) blood pressures between all participants with the balloon tubes facing down and up. Similarly, there were no significant differences in heart rates (*p* = 0.184). Figure 5 presents the Bland–Altman analyses of systolic and diastolic blood pressures measured by the cardiac monitor cuff with the balloon tubes facing down and up (*n* = 225).

**Table 4:** Blood pressure measurements with cardiac monitor cuffed balloon tubes facing downward and upward $\bar{x}$_ _±_ _s, using a paired samples *t*-test.

Population	Number of cases	Measurement site	SBP (mmHg)	DBP (mmHg)	Heart rate (beats/min)
All	225	Tube facing downward	121.91 ± 11.94	74.94 ± 9.86	75.05 ± 9.48
		Tube facing upward	122.04 ± 12.23	74.86 ± 10.37	74.81 ± 9.53
		Difference	−0.13 ± 2.56	0.08 ± 2.73	0.24 ± 2.65
		95% CI	−0.47 to 0.20	−0.2847 to 0.44	−0.1147 to 0.58
		*t*	−0.783	0.461	1.33
		*p*	0.435	0.645	0.184

Difference is the difference between blood pressure in the downward-facing position and the upward-facing position, *t*-value is the ratio of the downward-facing position to the upward-facing position, and *p*-value is the ratio of the downward-facing position to the upward-facing position.

Table 5 summarizes the measurement characteristics of four different populations, including sample size, age, height, weight, and BMI.

**Table 5:** Comparison of the anthropometric characteristics of the four groups.

Group	Number	Age	Male sex	Height	Weight	BMI
A	763	57.5 ± 10.1	446 (58.5%)	167.6 ± 8.3	62.1 ± 10.5	22.0 ± 2.8
B	253	58.4 ± 9.3	156 (61.7%)	169.1 ± 5.9	64.3 ± 8.5	23.0 ± 2.7
C	312	65.7 ± 11.4	167 (53.5%)	166.0 ± 6.9	61.6 ± 9.2	22.3 ± 2.4
D	225	62.2 ± 8.5	132 (58.7%)	166.8 ± 6.7	63.9 ± 10.1	22.9 ± 2.6

## Discussion

The precise measurement of blood pressure is fundamental to the diagnosis and treatment of hypertension (7). Currently, blood pressure is gauged through direct and indirect methods, with the former being less commonly employed due to its invasive nature. Indirect measurements involve occluding the brachial artery using a cuff and include methods such as mercury sphygmomanometers, which are based on the principle of Korotkoff sounds, and oscillometric sphygmomanometers, which rely on the principle of pulse wave detection (8). However, mercury sphygmomanometers are problematic due to issues such as environmental pollution and their relatively complex operation, leading to oscillometric sphygmomanometers being recommended as the preferred method for measuring blood pressure (9).

Blood pressure can be measured in various settings, including at clinics, in hospitals, at home, and through ambulatory monitoring. Nonetheless, irregularities in measurement techniques can lead to inaccuracies, potentially causing an overestimation of blood pressure, which can result in overdiagnosis and overtreatment, or an underestimation of blood pressure, which can delay necessary treatment (10, 11).

Many studies have examined the influence of different blood pressure measurement techniques on the results. Factors such as cuff length and width, the tightness of the cuff, rest time before measurement, sitting position, and the auscultation site of the stethoscope are well-established variables that can impact the accuracy of blood pressure readings (12–14).

Adhering to the 2017 ACC/AHA Hypertension Guidelines for Blood Pressure Measurement, the cuff's midpoint should be level with the right atrium (the midpoint of the sternum) during blood pressure assessment. Alternatively, the lower edge of the cuff should be located about 2–3 cm above the elbow crease. Despite this recommendation, previous studies have shown that only one-third of the population correctly positions their blood pressure cuffs (15). This is especially true for obese patients, who may find it difficult to place the cuff 2–3 cm above the elbow joint. With the prevalence of obesity rising globally, this challenge is magnified. In addition, in colder weather, particularly during winter, patients tend to wear excessive clothing. Rather than removing these layers to fully expose the upper arm, many roll up their sleeves, which can lead to incorrect cuff placement due to the difficulty of rolling up sleeves to the upper arm. This contributes to the common occurrence of misplacement of the cuff (16). Furthermore, individuals with thicker arms might only be able to measure their blood pressure at a lower position and may not realize that a larger cuff is required to ensure accurate readings (17).

In 1897, Hill and Barnard recognized the need for standardization in cuff positioning during blood pressure measurements, observing that blood pressure readings varied with changes in the cuff's distance from the level of the heart. They proposed that the expected change in blood pressure (Δbp) could be quantified by the formula Δbp = dv · *SG_b_*/*SG_m_* (12), where Δbp is the change in blood pressure as measured by the cuff in mmHg, dv is the vertical distance from the cuff to the right atrium in millimeters, *SG_b_* is the specific gravity of blood (1.05 at 37°C), and SGm is the specific gravity of mercury (approximately 13.6). This relationship is thus simplified to Δbp = (distance in millimeters/13.6 × 1.05) (18). According to this formula, for every 1 cm decrease in the distance between the cuff and the heart level, intra-arterial blood pressure is expected to increase by 0.8 mmHg, considering the specific gravities of blood and mercury. Given that the right atrium is located roughly at the level of the midpoint of the sternum (6), a cuff positioned closer to the feet will yield a lower blood pressure reading below the midpoint of the sternum, while a cuff closer to the head will yield a lower reading above this midpoint. These differences in blood pressure measurements are clinically significant, especially when considering the effects of antihypertensive medications (3).

The influence of cuff distance from the right atrium on blood pressure readings extends beyond theoretical concerns. Literature review has revealed at least two pertinent studies that confirm this effect (19, 20). These studies have specifically illustrated the impact of arm positioning on blood pressure by adjusting the arm's elevation, thus altering the distance from the cuff to the atria. In one study conducted by Mourad et al., hypertensive patients were randomly assigned to groups during ambulatory blood pressure monitoring. The experimental group had their blood pressure measured with their arm perpendicular to the torso, while the control group's measurements were taken with the arm parallel to the torso. The findings revealed that systolic blood pressure was 8.8 mmHg lower and diastolic blood pressure was 10.1 mmHg lower when the arm was perpendicular rather than parallel to the trunk. This study underscored the potential for significant variation in blood pressure readings based on arm position during measurement (19).

Mourad et al. also measured blood pressure using mercury and electronic sphygmomanometers. Individuals were initially positioned with their arms horizontally on a table, and then measurements were repeated with their arms parallel to the torso. This change in arm position led to an increase in mean blood pressure by 8/7 mmHg in normotensive participants and by 23/10 mmHg in hypertensive participants while sitting. These results were consistent with the proposed effects of arm positioning on blood pressure readings and highlighted the importance of proper cuff placement during the measurement process (19).

Kammila et al. observed that some patients experienced a significant nocturnal drop in blood pressure when ambulatory blood pressure monitoring data were analyzed. Upon further investigation, it was discovered that these patients had been sleeping with the hand being monitored on a pillow to muffle the sound of the ambulatory device and to increase comfort. Once this issue was identified, the patients were instructed to avoid using pillows for their arms at night and to keep their arms parallel to the bed. Following these instructions, the proportion of patients exhibiting a significant decrease in nocturnal systolic blood pressure dropped from 17.4% to 8.8%, and diastolic blood pressure from 37.0% to 24.4%. Conversely, the percentage of patients with an insignificant nocturnal decrease in blood pressure rose from 33.7% to 45.6% for systolic readings and from 13.0% to 31.6% for diastolic readings (20).

From the aforementioned findings, we can infer that the blood pressure readings obtained from a sphygmomanometer cuff vary depending on the position of the cuff, with readings being lower the further they are taken from the level of the heart.

Mercury sphygmomanometers, once the “gold standard” for in-office blood pressure measurements, have largely been replaced by automated sphygmomanometers. This shift occurred because mercury is recognized as a hazardous material. However, it is crucial for clinicians to be cognizant of the limitations presented by electronic sphygmomanometers in clinical settings. These devices may provide inaccurate readings in patients with cardiac arrhythmias, and not all electronic devices are reliable, effective, or suitable for every patient (21, 22). The mercury column sphygmomanometer operates on the principle that blood produces a Korotkoff sound as it flows through a constricted vascular space, creating a vortex. It comprises three main components: an inflatable cuff, a pressure gauge tube, and a mercury manometer. In the mercury manometer, the applied pressure acts directly on the brachial artery beneath the cuff. As the cuff deflates, the pressure on the brachial artery decreases, allowing blood flow to resume gradually. When the cuff pressure falls below the systolic pressure of the heart, blood flow is momentarily obstructed, creating a vortex and a detectable Korotkoff sound with a stethoscope. The reading on the sphygmomanometer at the moment this sound is first heard indicates the systolic blood pressure. The cuff is then slowly deflated further until the pressure equals the diastolic pressure of the heart, at which point the blood flow in the brachial artery becomes smooth, and the Korotkoff sound ceases. The sphygmomanometer reading at this moment represents the diastolic pressure (22, 23).

Some nursing textbooks in China state that the sphygmomanometer should be placed at the same height as the heart to ensure accurate blood pressure measurements. Is this truly necessary? Clinical experience from doctors and nurses shows that in situations where the patient cannot cooperate or where conditions are suboptimal or there is insufficient manpower, it is not easy for one person to maintain both the sphygmomanometer cuff and the device at the heart's level while also taking blood pressure readings. It would be convenient if the sphygmomanometer could be placed in any position, and in fact, the position of the sphygmomanometer has no effect on the measurement results. Both practice and theory can prove this point. When the sphygmomanometer cuff, the heart, and the mercury column zero point of the sphygmomanometer are at the same level, the pressure difference between the three is zero. However, if the mercury column zero point is higher or lower than the level of the cuff and the heart, there will be a pressure difference. Since the pressure between the cuff and the mercury column is transmitted by the air in the rubber tube connecting the cuff and the sphygmomanometer, the hydrostatic pressure formula, *P* = *ρ*gh (where *ρ* is the fluid density, g is the acceleration of gravity, and h is the fluid height or depth), applies. This formula suggests that the hydrostatic pressure is only related to the fluid height or depth for a given fluid medium, with density and gravitational acceleration being constants. Many assume that the height of the sphygmomanometer affects blood pressure values based on this principle. While the correctness of the formula is not in dispute, the conditions for its application have been overlooked; that is, it only applies to a fluid medium that is identical and continuous. The sphygmomanometer's cuff, when placed at the same level as the heart, is correct because the brachial artery is connected to the heart and contains blood, which meets the condition of the formula's sameness and continuity, leading to a correct conclusion. However, the construction of the sphygmomanometer involves three different fluids (blood, air, and mercury), which means the conditions of sameness and continuity for the formula are not met. The cuff is separated from the body, and the sphygmomanometer's mercury column interacts with air and mercury, breaking the continuity. Therefore, the perspective that the height of the sphygmomanometer affects brachial artery blood pressure readings is theoretically invalid. The correct approach is to apply the hydrostatic pressure formula in segments: *P* (brachial artery blood pressure) = *ρ* (mercury density) gh (height of the mercury column) ± *ρ* (air density) gh (height of the air column). With mercury having a density of 13,600 kg m^−3^ and air having a density of 1.29 kg m^−3^, it can be calculated that a change in the height of the sphygmomanometer (with the heart level as the zero point) by 1.054 m results in only a 1 mmHg change in the mercury column. This change is less than the error margin of two consecutive blood pressure measurements, indicating that a slight variation in the height of the sphygmomanometer has an imperceptible impact on blood pressure readings. In other words, the mercury sphygmomanometer can be placed at any height when measuring blood pressure.

The cuff of the electronic sphygmomanometer is placed in the same position and arm position as that of the mercury sphygmomanometer. By inflating and deflating the cuff, the blood flow in the brachial artery is altered to estimate the systolic and diastolic blood pressures. Human blood pressure varies with the time of day, with the maximum value being the systolic pressure and the minimum value being the diastolic pressure. The electronic sphygmomanometer measures these values—systolic and diastolic blood pressures (24). During the blood pressure measurement process, the cuff becomes pressurized, squeezing the blood vessels and causing an increase in blood pressure. This pressure peaks after deflation and the release of the cuff. As the cuff's pressure is continuously decreased, the blood vessels are gradually released, and the blood pressure lowers until it returns to normal. Throughout this process, the electronic sphygmomanometer cuff detects multiple small pulses generated by the blood vessel wall. The device's built-in program connects these pulses to form a curve known as the envelope. The device then analyzes and processes the envelope to automatically determine the diastolic and systolic blood pressures. Simultaneously, the shape of the envelope changes with the actual blood pressure, a principle known as the oscillometric method of blood pressure measurement (25). The oscillometric method relies on an oscillometric curve, making it less susceptible to interference from external noise, electrocautery, or other electronic surgical instruments. It transmits pressure through the gas in the cuff's tube, and the blood pressure measured is independent of the sphygmomanometer's placement; in other words, the sphygmomanometer can be placed at any height during the measurement.

In the fourth group of experiments, which is not mentioned in textbooks and guidelines, a certain cuff-tying method is suitable when the monitor is placed above the patient's head or on the bedside table. When the cuff is tied in reverse, that is, with the inflatable tube facing upward on the upper arm, it aligns straight with the monitor's blood pressure measuring tube, preventing it from bending or twisting. This reduces the risk of joint cracking and leakage, minimizes cuff wear, and ensures accurate blood pressure measurement, even when the patient bends at the elbow or moves their elbow joint. This method prevents the instrument from repeatedly inflating due to poor cuff inflation. When the patient is in a semi-recumbent position or has a flexed upper limb due to disease, the sphygmomanometer cuff can also be placed flat on the upper arm for the patient's comfort. However, clinical nurses using this method may face challenges from patients, peers, or be mistakenly perceived as performing irregular practices. It is hoped that the data from this clinical study will support the efficacy of this technique in actual clinical work.

We grouped the cardiac monitor cuffs with the balloon tubes facing different directions and performed blood pressure measurements, finding no significant differences in the measured values regardless of whether the cuffs had the balloon tubes facing up or down. For participants recruited from the cardiology department, the difference in mean blood pressure between cuffs with balloon tubes in the upward-facing position and in the standard cuff position was within 1.0 mmHg. Furthermore, blood pressure measured in the upward-facing position correlated positively with that in the standard position, and blood pressure concordance was good for all participants. On this basis, we conclude that the difference in mean blood pressure with the cuff's balloon tubing in both the upward and downward positions is within 1.0 mmHg, and that the differences in the measured outcomes are clinically acceptable. Similarly, since blood pressure measurements on cardiac monitors are made by the transfer of pressure through the gas in the cuff tube, the orientation of the cuff tube has a negligible effect on these measurements.

It is particularly important to emphasize that blood pressure is a variable hemodynamic phenomenon influenced by many factors (26). When we perform continuous blood pressure monitoring, the values are not completely consistent, and there are fluctuations known as blood pressure variability. This natural variation in blood pressure measurements is due to the patient's own status (26). Blood pressure variability increases when the patient is in an unstable state, thus enhancing the natural variation. A review of previous clinical studies showed that the natural variability of systolic blood pressure was in the range of 4–61 mmHg, and that of diastolic blood pressure was 2–39 mmHg (27). This suggests that the effects of changes in sphygmomanometer placement, cuff orientation, and balloon tube direction during blood pressure measurements are much smaller than those of natural variability and have a negligible impact on blood pressure measurements.

## Perspectives

This study contributes to the growing body of evidence that blood pressure measured by electronic sphygmomanometer cuffs placed in different positions varies, and that the farther away from the heart level, the lower the measured blood pressure value. Blood pressure measured at the elbow fossa position of upper-arm sphygmomanometers was lower than that at the standard upper-arm position by 3.48 mmHg systolic and 4.23 mmHg diastolic. Blood pressure readings from electronic and mercury sphygmomanometers are independent of the sphygmomanometer's position, and blood pressure readings from electronic sphygmomanometers are also independent of the orientation of the cuff and balloon tubing. Standardized blood pressure measurement can more accurately reflect a patient's actual blood pressure, aiding in active diagnosis and treatment, and significantly reducing the incidence of adverse cardiovascular events.

## Data Availability

The raw data supporting the conclusions of this article will be made available by the authors, without undue reservation.
